# Effects of Prone Positioning for Patients with Acute Respiratory Distress Syndrome Caused by Pulmonary Contusion: A Single-Center Retrospective Study

**DOI:** 10.1155/2022/4579030

**Published:** 2022-03-31

**Authors:** Xiaoyi Liu, Hui Liu, Shilian Liu, Wenlai Zhou, Qing Lan, Jun Duan, Xue Li, Xiangde Zheng

**Affiliations:** ^1^Department of Critical Care Medicine, The Central Hospital of Dazhou, Dazhou 635000, Sichuan, China; ^2^Ophthalmology, The Central Hospital of Dazhou, Dazhou 635000, Sichuan, China; ^3^Nosocomial Infection Control Department, The Central Hospital of Dazhou, Dazhou 635000, Sichuan, China; ^4^Department of Respiratory and Critical Care Medicine, The First Affiliated Hospital of Chongqing Medical University, Chongqing 400016, China; ^5^Department of Clinical Research Center, The First Affiliated Hospital of Chongqing Medical University, Chongqing 400016, China

## Abstract

**Background:**

The effects of prone positioning (PP) on patients with acute respiratory distress syndrome (ARDS) caused by pulmonary contusion (PC) are unclear. We sought to determine the efficacy of PP among patients whose ARDS was caused by PC.

**Methods:**

A retrospective observational study was performed at an intensive care unit (ICU) from January 2017 to June 2021. ARDS patients with PaO_2_/FiO_2_ (P/F) < 150 mmHg were enrolled. During the study period, we enrolled 121 patients in the PP group and 117 in the control group. The changes in vital signs, laboratory tests, and compliance of the respiratory system (Crs) were recorded for 3 consecutive days. The mechanical ventilation time, duration of ICU stay, complications, extubation rate, 28-day ventilator-free days, and mortality were also recorded.

**Results:**

In the PP group, the P/F and Crs increased over time. Compared to the control group, the P/F and Crs improved in the PP group over 3 consecutive days (*P* < 0.05). Furthermore, the PP group also had shorter total mechanical ventilation time (5.1 ± 1.4 vs. 9.3 ± 3.1 days, *P* < 0.05) and invasive ventilation time (4.9 ± 1.2 vs. 8.7 ± 2.7 days, *P* < 0.05), shorter ICU stay (7.4 ± 1.8 vs. 11.5 ± 3.6days, *P* < 0.05), higher extubation rate (95.6% vs. 84.4%, *P* < 0.05), less atelectasis (15 vs. 74, *P* < 0.05) and pneumothorax (17 vs. 24, *P* > 0.05), more 28-day ventilator-free days (21.6 ± 5.2 vs. 16.2 ± 7.2 days, *P* < 0.05), and lower mortality (4.4% vs. 13.3%, *P* < 0.05).

**Conclusions:**

Among PC cases with moderate to severe ARDS, PP can correct hypoxemia more quickly, improve Crs, reduce atelectasis, increase the extubation rate, shorten mechanical ventilation time and length of ICU stay, and reduce mortality.

## 1. Introduction

A pulmonary contusion (PC) is a parenchymal injury to the lung caused by an external force (e.g., trauma) either directly or indirectly acting on the chest [1]. It is often accompanied by clinical manifestations, including chest pain, chest tightness, shortness of breath, and hemoptysis. Often, in the most critically ill patients, the amount of pulmonary contusion may evolve into a severe acute respiratory distress syndrome (ARDS), i.e., severe hypoxemia, noncardiogenic pulmonary edema, and a decrease in pulmonary compliance. In the context of the current coronavirus disease 2019 (COVID-19) pandemic, the safe and effective management of PC patients has become more challenging [2], and the early elimination of COVID-19 can significantly improve the treatment of PC [3]. In recent years, the technical ability to establish a diagnosis of PC early and to predict disease aggravation has been significantly improved [4, 5], and a variety of clinical studies on the treatment of PC have been proven to be effective [6, 7], significantly reducing the clinical mortality of PC.

Prone positioning (PP) was first proposed as a treatment to improve ventilation in the 1970s [8]. A large number of studies have confirmed that PP can improve hypoxemia and prognosis in patients with ARDS [9, 10], but few reports have discussed PP treatment in patients with ARDS caused by PC [11]. Therefore, we retrospectively analyzed PP in the treatment of patients with ARDS caused by PC to verify the benefits of PP in this type of patient.

## 2. Materials and Methods

### 2.1. Subjects

A retrospective analysis was conducted of 371 PC patients admitted to the intensive care unit (ICU) of the Central Hospital of Dazhou, China, from January 2017 to July 2021. In all, 264 patients were screened according to the inclusion and exclusion criteria. Of these, 136 patients received PP treatment and 128 did not. The total group included 197 males and 67 females, aged from 16 to 74 years old. Upon entry to the ICU, each patient, to receive a diagnosis of PC, was required to exhibit the following indicators: an Acute Physiology and Chronic Health Score (APACHE II) of 12 to 32 points, an Injury Severity Score (ISS) of 14 to 32 points, a platelet count of 159 to 290 × 10^9^/L, a hemoglobin value of 82 to 142 g/L, a heart rate (HR) of 87 to 136 beats/min, a mean arterial pressure (MAP) of 62 to 83 mmHg, a respiratory rate (RR) of 17 to 32 beats/min, an arterial blood gas pH of 7.332 to 7.492, an arterial blood carbon dioxide partial pressure (PaCO_2_) of 35.6 to 43.8 mmHg, and an oxygenation index (P/F) of 126 to 236 mmHg, as well as a chest X-ray or chest CT scan that allowed a diagnosis of PC.

### 2.2. Inclusion and Exclusion Criteria

#### 2.2.1. Inclusion Criteria

The inclusion criteria were as follows: (1) ARDS caused by PC treated with invasive mechanical ventilation; (2) diagnosis of ARDS meeting the 2012 Berlin definition of ARDS [12]; (3) the occurrence of moderate to severe ARDS, P/F < 150 mmHg [13]; and (4) an age of 16–75 years old.

#### 2.2.2. Exclusion Criteria

The exclusion criteria were as follows: (1) noninvasive ventilation support performed before orotracheal intubation; (2) previous lung diseases; (3) pelvic, cervical, or spinal fracture or requiring a fixed position; (4) uncontrolled increase in intracranial pressure; (5) open abdominal injury; (6) multiple traumas with unstable fractures; (7) pregnancy; and (8) severe hemodynamic instability (mean arterial blood pressure <60 mmHg or systolic blood pressure >200 mmHg) [14].

### 2.3. Grouping

**Figure 1:**
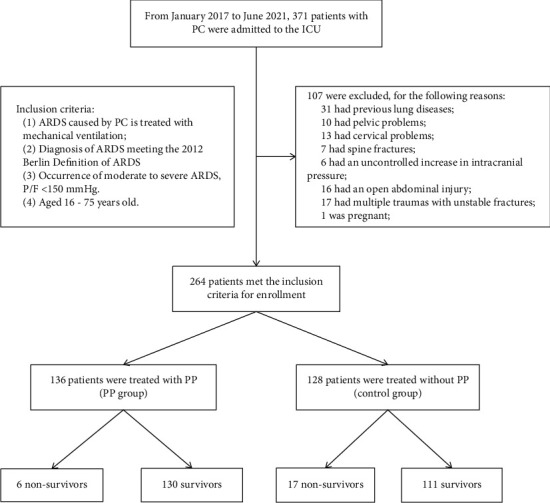
Patient-screening flowchart.

The 335 PC patients admitted to the ICU were screened according to the inclusion and exclusion criteria, and 264 cases were included in the study. The included cases were divided into a PP group (*n* = 136) and a control group (*n* = 128) (Figure 1) according to whether they were treated with PP to alleviate severe hypoxemia when the P/F was lower than 150 mmHg [13, 15]. The treatment of the patients followed the ARDS treatment principle [13]. First, bromhexine hydrochloride was injected (4 mg three times a day, intravenous drip) as an expectorant. Then, tanreqing was injected (40 mL once a day, intravenous drip) to clear away heat and resolve phlegm. Next, select antibiotics were administered to fight infection according to the results of pathogenic microorganism culture and the antibacterial spectrum. Fourth, sufentanil citrate was injected (0.2 *μ*g/[kg•h], intravenous pumping) as anesthesia. Fifth, midazolam was injected (0.3 *μ*g/[kg•h], intravenous pumping) as a sedative. Sixth, the patient was fed with full-tube nutrition support. Next, we determined the appropriate mechanical ventilation strategy with low tidal volume lung protective ventilation (LTV), and the positive-end expiratory pressure (PEEP) and inspiratory oxygen fraction (FiO_2_) values (lower PEEP/higher FiO_2_) according to the ARDSnet protocol [16–18]. Finally, lung recruitment was conducted with pressure-controlled ventilation using a recruiting maneuver [19]. Other treatments such as using vibration to release sputum, bronchoscopy, maintenance of internal environment stability, prophylactic noninvasive ventilation immediately after extubation, and intensive care were performed when necessary. The ventilator was adjusted according to the arterial blood gas results until the patient was evacuated from the ventilator. In controls, the arterial blood gas was collected in the supine position, and in the PP group, it was collected in the supine position after the end of PP. Before measuring the compliance of the respiratory system (Crs) in both groups, we confirmed adequate sedation and restrained the patients from breathing spontaneously. When analgesia and tranquilizers did not effectively inhibit spontaneous respiration or were limited by negative hemodynamic effects, short-acting neuromuscular blockers were used, if necessary, as standard measurement indicators. In the PP group, when the P/F was lower than 150 mmHg, PP was used to treat patients with severe hypoxemia until the P/F > 200 mmHg could be sustained and then the PP treatment was stopped; the PP time was 12–16 h/day [16]. During PP, patients with chest and abdomen trauma were bound and fixed with medical chest straps, and the area surrounding the trauma was raised using a water bag such that the traumatic site could be elevated. Changes in arterial blood gas and Crs were observed for 3 consecutive days after enrollment in the two groups. Three days after enrollment, a chest CT was performed to assess atelectasis [13]. The statistics for the total mechanical ventilation time (including invasive ventilation time and noninvasive ventilation time), invasive ventilation time, noninvasive ventilation time, ICU stay, atelectasis, pneumothorax, extubation rate, and mortality of the two groups were collected.

### 2.4. Ethics

Informed consent was obtained from the patients or their family members following medical regulations and routines. Our ethics committee reviewed and approved the study protocol (No. 2020010).

### 2.5. Statistical Analyses

SPSS 22.0 statistical software was used for statistical analyses. The measurement data were tested using a *t*-test and are expressed as means ± standard deviations. Categorical variables were tested using chi-square tests. The independent risk factors related to death were analyzed using multivariate logistic regression. *P* < 0.05 was considered statistically significant.

## 3. Results

### 3.1. Patient Characteristics

There were no statistically significant differences in basic information between the two groups of patients upon entry to the ICU in terms of age, gender, APACHE II score, ISS, basic diseases, combined trauma site, complications, vital signs, or laboratory examinations (Table 1). The average stay in the ICU before the PP treatment of the PP group was (10.1 ± 3.5) h.

### 3.2. Changes in Arterial Blood Gas and Crs

**Figure 2:**
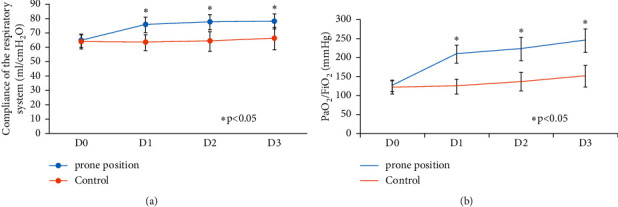
Changes in compliance of the respiratory system and PaO_2_/FiO_2_. (a) Trend of change in compliance of the respiratory system; (b) trend of change in PaO_2_/FiO_2_.

At enrollment, there were no significant differences in arterial blood gas results (pH, PaCO_2_, P/F, FiO_2_) or Crs between the two groups. One day later, these parameters for controls were not statistically different compared to day 1. However, P/F (125.8 ± 15.6 vs. 208.9 ± 23.1 mmHg, *P* < 0.05) and Crs (64.7 ± 4.8 vs. 75.8 ± 5.4 mL/cmH_2_O, *P* < 0.05) significantly increased and FiO_2_ (0.99 ± 0.04 vs. 0.78 ± 0.08, *P* < 0.05) decreased in the PP group; pH and PaCO_2_ did not significantly change. There were significant differences in P/F and Crs that remained higher and FiO_2_ lower in the PP group than in controls for all 3 consecutive days following enrollment (Figure 2), but the differences in pH and PaCO_2_ were not statistically significant (Table 2).

### 3.3. Independent Risk Factors Associated with Death

Multivariate logistic regression analysis of independent risk factors was performed for death on gender, age, ISS score, APACHE II score, platelets, hemoglobin, basic diseases, arterial blood gas at ICU entry, arterial blood gas when enrolled in the study, and PP. PP was found to be a protective factor (odds ratio = 0.004, 95% CI: 0.00–0.11; Table 3).

### 3.4. Outcomes

After 3 days of enrollment, chest CT showed an increase in the incidence of pneumothorax and atelectasis in the two groups, but the incidences of both were lower in the PP group than in the control group. A comparison of the two groups of patients after treatment showed that the PP group had shortened total mechanical ventilation time, invasive ventilation time, and noninvasive ventilation time, shorter ICU stay, fewer patients on prophylactic noninvasive ventilation after extubation, higher extubation rate, more 28-day ventilator-free days, as well as lower mortality. With the exception of that for pneumothorax, the differences in all observation indicators were statistically significant (Table 4).

## 4. Discussion

PC is always followed by an accumulation of interstitial fluid and decreased alveolar membrane diffusion function, which may lead to ARDS [20]. Studies have shown that ARDS is an important factor leading to the late clinical death of PC patients [5, 21]. PP is an effective measure for the treatment of ARDS, but there has not yet been a clinical study on the efficacy of PP in the treatment of ARDS caused by PC. In our clinical study, we found that PP can safely be used in patients with ARDS caused by PC, as it improves hypoxemia and Crs and has a positive effect on the outcome of treatment.

Most PC patients show chest and abdomen trauma, which increases the difficulty of PP implementation and the risk of complications, limiting the clinical implementation of PP. Therefore, there has been no clinical research on PP treatment of patients with PC after ARDS. We used elastic chest straps to address the wounds of our patients to prevent secondary injuries caused by wound dehiscence during PP. At the same time, the surrounding area of the wound was raised using water bags to elevate the injured part and avoid sores caused by prolonged pressure. Our results showed that the trauma sites were not aggravated and did not acquire pressure sores during PP in the PP group, confirming that this procedure can be safely used in the clinical treatment of PC patients with ARDS.

Our retrospective analysis found that the P/F and Crs significantly increased and FiO_2_ decreased in the PP group after 1 day of treatment, and the clinical indicators continued to improve over 3 consecutive days. At an average of 3.0 ± 1.1 days after PP treatment, P/F > 200 mmHg could be continuously maintained, and the clinical effect was better than that for the control group. PP can change gravity-dependent lung areas by changing the body position, improving the ventilation/blood flow ratios of patients with moderate to severe ARDS, improving Crs, and quickly correcting early hypoxemia.

PC patients often develop pulmonary ARDS, showing poor lung recruitment and poor response to conventional lung recruitment techniques, resulting in a high incidence of atelectasis [22]. In this study, we used chest CT to assess atelectasis, the incidence of which was 57.8% in the control group and only 11.0% in the PP group 3 days after enrollment. PP changes the gravity-dependent area of the lung, which can prevent consolidation of such areas in ARDS patients; this has the effect of continuous lung opening to achieve lung recruitment and reduce the occurrence of atelectasis during the treatment process. The use of LTV for mechanical ventilation can reduce the risk of barotrauma.

Studies have shown that PP treatment can improve hypoxia and Crs while also controlling inflammatory factors in the lungs [23]. In our study, the PP group had a shorter total mechanical ventilation time, invasive ventilation time, and noninvasive ventilation time; a higher extubation rate; fewer (14%) patients receiving prophylactic noninvasive ventilation after extubation; shorter ICU stay; and a lower mortality rate. The most plausible explanation is that PP quickly corrected the patient's hypoxemia and reduced the ventilator-related lung damage and release of proinflammatory cytokines, the time of tracheal intubation, and the risk of exposure to ventilator-associated pneumonia. At the same time, the postural drainage of PP has a certain airway clearance effect and partly reduces the risk of disease deterioration. Therefore, the patients in the PP group had shorter mechanical ventilation time, a higher extubation rate, and fewer patients with prophylactic noninvasive ventilation after extubation than those without PP treatment. PP treatment also increased the number of 28-day ventilator-free days and reduced the mortality rate. In sum, PP had a positive effect on the treatment of patients with ARDS caused by PC.

There have been many studies on PP and its performance for the treatment of critical ARDS, in which it has been shown that the clinical efficacy is affected by multiple factors [24]. In our study, better curative effects were observed for the patients in the PP group. The possible reasons for this include the absence of lung infection in early ARDS caused by PC, which is generally a low-inflammation type of ARDS that responds well to PP treatment.

This study had several limitations. First, it was a clinical retrospective study of ARDS caused by PC, and its retrospective nature is a potential cause for bias. However, we conducted a multivariate logistic regression analysis of independent risk factors for death, which showed that PP can affect treatment outcomes. Second, the subjects were ICU patients with severe disease, and their treatment measures were complex. Third, the sample size was small, the data collection was relatively simple, and the retrospective design only provided low-quality evidence. Therefore, a randomized controlled trial with larger samples is required to provide high-quality evidence.

## 5. Conclusions

For patients showing PC with ARDS (P/F < 150 mmHg), PP can change the gravity-dependent area of the lung, thereby improving lung ventilation/blood flow ratios, correcting early hypoxemia in a timely fashion, and improving Crs. It can also reduce the occurrence of atelectasis, increase the extubation rate, shorten the duration of mechanical ventilation and ICU stay, increase the number of 28-day ventilator-free days, and reduce mortality rates, while maintaining the safety required for clinical application.

## Figures and Tables

**Table 1:** Demographic, clinical, and laboratory findings upon entry to the ICU.

	Total (*n* = 264)	PP (*n* = 136)	Control (*n* = 128)	Difference (95% CI)	*P* value
Age, years	54.7 ± 14.8	54.1 ± 16.3	55.3 ± 13.1	1.2 (−2.4, 4.8)	0.499
Gender					
Female	67 (25.4%)	35 (25.7%)	32 (25.0%)	—	0.891
Male	197 (74.6%)	101 (74.3%)	96 (75.0%)	—	0.891
APACHE II score	21.9 ± 3.2	22.2 ± 2.8	21.6 ± 3.6	−0.6 (−1.4, 0.2)	0.129
ISS score	20.5 ± 3.8	20.5 ± 4.0	20.4 ± 3.6	−0.0 (−1.0, 0.9)	0.957
Basic diseases					
Hypertension	16 (6.1%)	10 (7.4%)	6 (4.7%)	—	0.364
Diabetes	31 (11.7%)	17 (12.5%)	14 (10.9%)	—	0.693
Chronic renal insufficiency	7 (2.7%)	3 (2.2%)	4 (3.1%)	—	0.642
Chronic liver insufficiency	8 (3.0%)	5 (3.7%)	3 (2.3%)	—	0.528
Combined trauma site					
Head	79 (29.9%)	41 (30.2%)	38 (29.7%)	—	0.935
Chest	197 (74.6%)	101 (74.3%)	96 (75.0%)	—	0.891
Abdomen	64 (24.2%)	36 (26.5%)	28 (21.9%)	—	0.384
Limbs	78 (29.6%)	37 (27.2%)	41 (32.0%)	—	0.390
Clinical complications					
Pulmonary hemorrhage	24 (9.1%)	13 (9.6%)	11 (8.6%)	—	0.785
Pneumothorax	25 (9.5%)	15 (11.0%)	10 (7.8%)	—	0.372
Atelectasis	12 (4.6%)	7 (5.2%)	5 (3.9%)	—	0.629
Heart rate, beats/min	113.6 ± 9.8	114.4 ± 9.6	112.7 ± 9.9	−1.8 (−4.1, 0.6)	0.146
MAP, mmHg	71.1 ± 4.9	71.5 ± 4.9	70.7 ± 4.8	−0.9 (−2.1, 0.3)	0.147
Respiratory rate, breaths/min	23.2 ± 3.8	23.1 ± 3.9	23.3 ± 3.6	0.2 (−0.8, 1.1)	0.736
Hb, g/L	118.7 ± 13.0	118.3 ± 13.5	119.1 ± 12.6	0.9 (−2.3, 4.1)	0.576
Platelet count, 10^9^/L	218.0 ± 24.0	219.8 ± 21.4	216.1 ± 26.4	−3.68 (−9.49, 2.12)	0.213
pH	7.44 ± 0.05	7.44 ± 0.05	7.44 ± 0.05	0.00 (−0.01, 0.01)	0.883
PaCO_2_, mmHg	40.2 ± 2.0	40.7 ± 1.7	39.7 ± 2.1	−0.97 (−1.44, −0.50)	0.107
P/F, mmHg	172.5 ± 27.1	171.6 ± 26.5	173.3 ± 27.9	1.67 (−4.91, 8.26)	0.617
FiO_2_	0.5 ± 0.1	0.5 ± 0.1	0.5 ± 0.1	0.09 (−0.01, 0.03)	0.299

APACHE II, acute physiology and chronic health evaluation II; CI, confidence interval; FiO_2,_ inspiratory oxygen fraction; Hb, hemoglobin; ISS, injury severe score; MAP, mean arterial pressure; PaCO_2_, arterial partial pressure of carbon dioxide; P/F, partial pressure of oxygen in arterial blood/fraction of inspired oxygen; PP, prone position.

**Table 2:** Changes in arterial blood gas and Crs.

	PP (*n* = 136)	Control (*n* = 128)	Difference (95% CI)	*P* value
When enrolled in the study				
pH	7.44 ± 0.05	7.44 ± 0.04	0.00 (−0.11, 0.01)	0.559
PaCO_2_, mmHg	38.4 ± 1.7	39.1 ± 1.9	0.7 (0.3, 1.2)	0.163
P/F, mmHg	125.8 ± 15.6^＃^	121.9 ± 18.1	−3.9 (−8.0, 0.2)	0.061
FiO_2_	0.99 ± 0.04^＃^	0.98 ± 0.06	−0.008 (−0.020, 0.004)	0.198
Crs, mL/cmH_2_O	64.7 ± 4.8^＃^	63.7 ± 5.0	−1.0 (−2.2, 0.2)	0.109
1 day after enrollment				
pH	7.43 ± 0.05	7.44 ± 0.05	0.01 (−0.00, 0.01)	0.199
PaCO_2_, mmHg	39.3 ± 1.4	39.2 ± 1.4	−0.1 (−0.4, 0.3)	0.959
P/F, mmHg	208.9 ± 23.1	125.0 ± 19.2	−80.87 (−89.04, −78.71)	<0.001^*∗*^
FiO_2_	0.78 ± 0.08	0.98 ± 0.05	0.195 (0.179, 0.212)	<0.001^*∗*^
Crs, mL/cmH_2_O	75.8 ± 5.4	63.4 ± 5.5	−12.5 (−13.8, −11.1)	<0.001^*∗*^
2 days after enrollment				
pH	7.43 ± 0.05	7.45 ± 0.04	0.02 (0.01, 0.03)	0.377
PaCO_2_, mmHg	39.4 ± 1.4	39.2 ± 1.5	−0.2 (−0.6, 0.2)	0.394
P/F, mmHg	222.6 ± 30.8	136.7 ± 24.1	−85.9 (−92.6, −79.2)	<0.001^*∗*^
FiO_2_	0.58 ± 0.09	0.95 ± 0.09	0.367 (0.346, 0.389)	<0.001^*∗*^
Crs, mL/cmH_2_O	77.6 ± 5.3	64.2 ± 7.8	−13.4 (−14.9, −11.9)	<0.001^*∗*^
3 days after enrollment				
pH	7.43 ± 0.05	7.45 ± 0.04	0.02 (0.01, 0.03)	0.684
PaCO_2_, mmHg	39.3 ± 1.3	39.5 ± 1.4	0.02 (−0.31, 0.35)	0.282
P/F, mmHg	245.4 ± 30.9	152.2 ± 28.6	−93.3 (−100.5, −86.1)	<0.001^*∗*^
FiO_2_	0.51 ± 0.1	0.79 ± 0.17	0.284 (0.250, 0.317)	<0.001^*∗*^
Crs, mL/cmH_2_O	78.1 ± 5.3	66.1 ± 7.6	−11.99 (−13.6, −10.4)	<0.001^*∗*^

Crs, compliance of the respiratory system; CI, confidence interval; FiO_2,_ inspiratory oxygen fraction; PaCO_2_, arterial partial pressure of carbon dioxide; P/F, partial pressure of oxygen in arterial blood/fraction of inspired oxygen; PP, prone position. ^#^*P*  <  0.05 for comparisons between enrollment and after 1 day of enrollment. ^*∗*^*P*  <  0.05 for patients with versus without PP. 1 cmH_2_O = 0.098 kPa.

**Table 3:** Independent risk factors associated with death in hospital identified by multivariate logistic regression analysis.

Risk factors	OR (95% CI)	*P* value
ISS score	3.014 (1.68, 5.40)	<0.001^*∗*^
PP	0.004 (0.00, 0.11)	0.001^*∗*^
P/F when enrolled in the study, mmHg	0.907 (0.84, 0.98)	0.009^*∗*^

CI, confidence interval; ISS, injury severe score; OR, odds ratio; P/F, partial pressure of oxygen in arterial blood/fraction of inspired oxygen; PP, prone position. ^*∗*^*P*  <  0.05 for patients with versus without PP.

**Table 4:** Outcomes of mechanical ventilation, ICU stay, clinical complications, and mortality.

	Total (*n* = 264)	PP (*n* = 136)	Control (*n* = 128)	Difference (95% CI)	*P* value
Total mechanical ventilation time^#^, days	7.1 ± 3.2	5.1 ± 1.4	9.3 ± 3.1	4.2 (3.6, 4.8)	<0.001^*∗*^
Invasive ventilation time, days	6.8 ± 2.8	4.9 ± 1.2	8.7 ± 2.7	3.8 (3.3, 4.3)	<0.001^*∗*^
Number of prophylactic noninvasive ventilations after extubation	52 (19.7%)	19 (14.0%)	33 (25.8%)	—	0.016^*∗*^
Noninvasive ventilation time, days	0.4 ± 0.8	0.2 ± 0.5	0.5 ± 0.9	0.3 (0.1, 0.5)	0.001^*∗*^
Extubation rate, %	238 (90.2%)	130 (95.6%)	108 (84.4%)	—	0.002^*∗*^
28-day ventilator-free, days	19.0 ± 6.8	21.6 ± 5.2	16.2 ± 7.2	−5.4 (−7.0, −3.9)	<0.001^*∗*^
ICU stay time before enrollment, hours	10.6 ± 3.8	10.1 ± 3.5	11.2 ± 4.0	1.1 (0.2, 2.0)	0.157
PP time, days	1.5 ± 1.7	3.0 ± 1.1	0	3.0 (2.8, 3.2)	<0.001^*∗*^
ICU stay time, days	9.4 ± 3.5	7.4 ± 1.8	11.5 ± 3.6	4.2 (3.5, 4.8)	<0.001^*∗*^
Clinical complications (3 days after enrollment)					
Pneumothorax	41 (15.5%)	17 (12.5%)	24 (18.8%)	—	0.161
Atelectasis	90 (34.1%)	15 (11.0%)	74 (57.8%)	—	<0.001^*∗*^
Mortality, %	23 (7.7%)	6 (4.4%)	17 (13.3%)	—	0.011^*∗*^

CI, confidence interval; ICU, intensive care unit; PP, prone position. ^#^Total mechanical ventilation time includes the time of invasive and noninvasive mechanical ventilation. ^*∗*^*P*  <  0.05 for patients with versus without PP.

## Data Availability

The data are available from the corresponding author on reasonable request.

## Abbreviations

APACHE II: Acute Physiology and Chronic Health Evaluation II
ARDS: Acute respiratory distress syndrome
Crs: Compliance of respiratory system
CI: Confidence interval
FiO_2_: Inspiratory oxygen fraction
Hb: Hemoglobin
ICU: Intensive care unit
ISS: Injury severe score
LTV: Low tidal volume lung protective ventilation
MAP: Mean arterial pressure
OR: Odds ratio
PaO_2_: Arterial partial pressure of oxygen
PaCO_2_: Arterial partial pressure of carbon dioxide
PC: Pulmonary contusion
PEEP: Positive-end expiratory pressure
P/F: Partial pressure of oxygen in arterial blood/fraction of inspired oxygen
PP: Prone positioning.
